# Airspace dimension assessment with nanoparticles as a proposed biomarker for emphysema

**DOI:** 10.1136/thoraxjnl-2020-214523

**Published:** 2021-04-15

**Authors:** H Laura Aaltonen, Madeleine Petersson Sjögren, Jonas K F Jakobsson, Hanna Nicklasson, Sandra Diaz, Francisco Sánchez Montiel, Sophia Zackrisson, Veronica Ideböhn, Gunnar Engström, Jakob Löndahl, Per Wollmer

**Affiliations:** 1 Department of Translational Medicine, Diagnostic Radiology, Lund University, Lund, Sweden; 2 Department of Imaging and Functional Medicine, Skåne University Hospital, Malmö, Sweden; 3 Department of Radiology, University of Washington, Seattle, Washington, USA; 4 Department of Design Sciences, Lund University, Lund, Sweden; 5 Lund University, NanoLund, Lund, Sweden; 6 Department of Pediatric Radiology, Karolinska University Hospital, Stockholm, Sweden; 7 Department of Clinical Sciences, Lund University, Malmö, Sweden

**Keywords:** emphysema, respiratory measurement, exhaled airway markers, imaging/CT MRI

## Abstract

Airspace dimension assessment with nanoparticles (AiDA) is a novel method to measure distal airspace radius non-invasively. In this study, AiDA radii were measured in 618 individuals from the population-based Swedish CArdiopulmonary BioImaging Study, SCAPIS. Subjects with emphysema detected by computed tomography were compared to non-emphysematous subjects. The 47 individuals with mainly mild-to-moderate visually detected emphysema had significantly larger AiDA radii, compared with non-emphysematous subjects (326±48 µm vs 291±36 µm); OR for emphysema per 10 µm: 1.22 (1.13–1.30, p<0.0001). Emphysema according to CT densitometry was similarly associated with larger radii compared with non-emphysematous CT examinations (316±41 µm vs 291 µm±26 µm); OR per 10 µm: 1.16 (1.08–1.24, p<0.0001). The results are in line with comparable studies. The results show that AiDA is a potential biomarker for emphysema in individuals in the general population.

## Introduction

Chronic obstructive pulmonary disease (COPD) originates in the distal airspaces, causing chronic inflammation and irreversible airspace enlargement, emphysema. The emphysematous component of COPD can be diagnosed by CT, which may be poorly accessible, expensive and complicated by large interobserver variation in interpretation, especially at early stages of disease. Reduced diffusing capacity for carbon monoxide (*D*_L, CO_), in the presence of airflow obstruction, is indicative of emphysema, but the method is not specific.^1^

We have suggested a simple method, airspace dimension assessment (AiDA), to determine distal airspace radius based on inhalation of nanoparticles. Nanoparticles deposit in the distal airspaces by diffusion, the probability being dependent on residence time in the lung and distance to an airspace wall. Measurement of deposition related to time allows the mean airspace radius (*r*_AiDA_) to be calculated.^2–5^ In a proof-of-concept study, a preliminary version of the method differentiated emphysema patients from healthy controls.^6^

The aim of this study was to determine if *r*_AiDA_ differs between persons with and without CT-verified emphysema in an unselected population. We expected persons with enlarged, emphysematous airspaces to have larger *r*_AiDA_ compared with non-emphysematous individuals. Secondary and tertiary aims were to determine whether subjects with emphysema suggested by lung function parameters have larger *r*_AiDA_ relative to non-emphysematous persons, and to investigate the role of comorbidities.

## Methods

**Figure 1:**
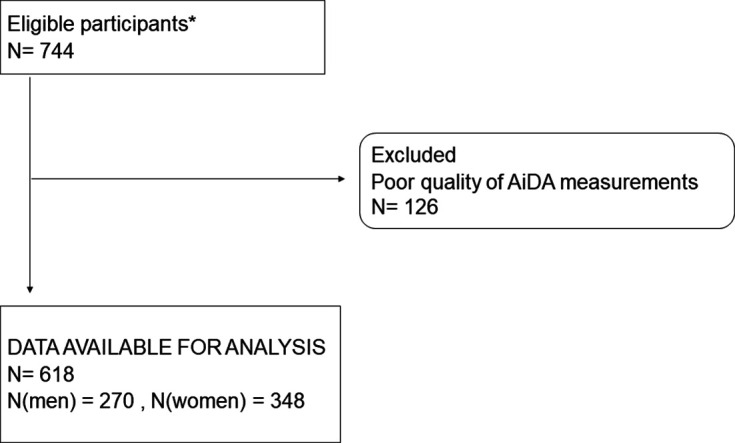
Exclusion chart. *Within the municipality of Malmö, Sweden, there were 51 061 registered inhabitants in the target age group in 2015. During the study time period, 4716 randomly selected individuals from the population registry were contacted, of which 50% (2358) participated. Of these, 744 randomly selected subjects underwent the AiDA measurements, corresponding to 1.5% of the target age population within the municipality. Please see online supplemental 1 for successful measurement criteria. AiDA, Airspace dimension assessment with nanoparticles.

The Swedish CArdioPulmonary bioImage Study (SCAPIS) is a national population-based study with 30 154 participants between 50 and 64 years of age. Our study was performed in a random sample of participants examined in Malmö, Sweden, between 2014 and 2016 (figure 1, online supplemental 1).

10.1136/thoraxjnl-2020-214523.supp1 Supplementary data

In AiDA measurements, the subjects inhaled 50 nm nanoparticles and held their breath for 5–10 s. Exhaled nanoparticles were measured from a sample at a volumetric lung depth of 1300 mL. The procedure was repeated six times. Particle recovery was calculated as the ratio between exhaled and inhaled concentration.^3^ An exponential decay curve was fitted to the recovery values obtained at different breath-hold times, and the half-life (*t*_½_) was calculated. By solving the diffusion equation, rAiDA is obtained:

$$r_{AiDA}=2.89\sqrt{D}t_{½}$$

where *D* is the diffusion coefficient given by the Stokes-Einstein equation.^2^

A chest CT was obtained and interpreted visually by one of four chest radiologists. A semiquantitative emphysema score with a maximum value of 18 was recorded (online supplemental 1). CT-derived total lung capacity by volumetric CT was calculated, and the percentage of voxels with a Hounsfield unit value below −950 (RV-950) was recorded. Emphysema was also defined quantitatively using two RV-950 percentage thresholds; >7% and >5%. Pulmonary function tests were performed according to American Thoracic Society and European Respiratory Society (ATS/ERS) standards.

## Results

Of the 744 subjects who underwent AiDA measurements, 618 were eligible for analysis (figure 1). The 47 persons with visually detected emphysema demonstrated an average emphysema score of 3.4±3.2, indicating mild-to-moderate disease. Most subjects had normal lung function, but some showed airflow obstruction. The *r*_AiDA_ was approximately normally distributed (online supplemental 3).

10.1136/thoraxjnl-2020-214523.supp2 Supplementary data

The persons with emphysema had a significantly larger *r*_AiDA_ compared with non-emphysematous subjects (tables 1A, B). By visual CT interpretation, the mean difference was 35 µm (95% CI 21 to 50 µm, p<0.0001). Findings were similar for emphysema defined by CT densitometry; mean differences were 25 µm (95% CI 11 to 36 µm, p<0.0001) and 37 µm (95% CI 15 to 59 µm, p<0.0001) for the 5% and 7% thresholds, respectively.

**Table 1A:** Subject characteristics with and without visually detected emphysema

	Absent				Present				T-test
	N	M	SD	Range	N	M	SD	Range	P value
Age (year)	563	57.3	4.5	50–65	47	59.2	4.2	51–65	0.004
Weight (kg)	563	80	16	43–146	47	81	17	53–121	NS
Height (cm)	563	171	9	146–199	47	172	10	158–194	NS
BMI (kg/m^2^)	563	27	5	17–45	47	27	4	18–36	NS
TLC (CTV) (L)	493	5.3	1.3	2.3–10.1	40	6.0	1.5	4.0–10.0	0.006
VC (L)	561	4.0	0.9	2.1–6.5	46	3.9	1.1	1.9–7.3	NS
VC (% pred)	561	110	15	66–154	47	107	16	60–143	NS
FEV_1_ (L)	561	3.1	0.70	1.55–5.35	47	2.7	0.91	0.99–5.35	0.006
FEV_1_ (% pred)	561	107	14	65–152	47	93	22	30–138	<0.0001
D_L, CO_ (mmol min^-1^ kPa^-1^)	530	8.12	1.61	4.47–14.66	45	7.16	2.20	2.64–12.82	0.006
D_L, CO_ (% pred)	526	91	13	54–170	43	81	20	29–134	0.001
RV −950 (%)	493	1.9	1.9	0–11	40	2.8	4.3	0–23	NS
Pack-years	517	9.9	12.8	0–86	44	27.6	16.0	0–66	<0.0001
r_AiDA_ (µm)	563	291	36	214–428	47	326	48	266–516	0.00001

**Table 1B:** Subject characteristics with and without emphysema according to CT RV-950 cutoff >5%

	Absent				Present				T-test
	N	M	SD	Range	N	M	SD	Range	P value
Age (y)	492	57.4	4.5	50–65	41	57.5	4.7	50–65	NS
Weight (kg)	492	80	16	43–139	41	89	13	54–106	NS
Height (cm)	492	171	9	146–199	41	177	9	151–197	<0.0001
BMI (kg/m^2^)	492	27	5	17–45	41	25	4	18–34	0.01
TLC (CTV) (L)	492	5.2	1.2	2.3–10.1	41	7.1	9.4	5.5–9.2	<0.0001
VC (L)	492	3.9	0.9	1.9–7.3	40	4.8	0.9	2.5–6.4	<0.0001
VC (% pred)	491	110	15	60–154	40	112	13.2	78–139	NS
FEV_1_ (L)	491	3.1	0.72	0.27–5.35	40	3.45	0.95	0.99–5.08	0.03
FEV_1_ (% pred)	491	106	15	46–152	40	103	22	30–139	NS
D_L, CO_ (mmol min^-1^ kPa^-1^)	463	8.07	1.67	2.2–14.3	38	8.49	1.93	2.6–11.3	NS
D_L, CO_ (% pred)	460	91	13	42–170	38	89	17	29–117	NS
RV −950 (%)	492	1.4	1.2	0–5	41	7.4	5.2	5–23	
Pack-years	453	10.5	14.2	0–86	38	10.1	14.8	0–54	NS
r_AiDA_ (µm)	492	291	36	214–516	41	316	41	239–412	<0.0001

AiDA, Airspace dimension assessment with nanoparticles; BMI, body mass index; TLC (CTV), total lung capacity measured by volumetric CT; D_LCO_, diffusing capacity for carbon monoxide; FEV_1_, forced expiratory flow in one second;NS, not significant; r_AiDA_, distal airspace radius measured with the AiDA method; RV-950, the relative volume of voxels in lung parenchyma with a Hounsfield Unit value less than -950; TLC, total lung capacity; VC, vital capacity.

Dividing the *r*_AiDA_ into tertiles, we observed that with increasing radius, an increasing percentage of the subjects had emphysema and airflow obstruction. (online supplemental 4)

10.1136/thoraxjnl-2020-214523.supp4 Supplementary data

Logistic regression analysis was conducted using several definitions of emphysema and airflow obstruction (table 2). The radius was associated with increased OR with little effect of adjustments. No comorbidities caused significant differences in *r*_AiDA_ (online supplementals 1 and 2).

10.1136/thoraxjnl-2020-214523.supp3 Supplementary data

**Table 2:** r_AiDA_ logistic regression models; odds ratios (95% CIs) N=618

	N	Model 1 OR	Model 2 OR	Model 3 OR
Emphysema present in CT, visual evaluation	47	1.216 (1.134–1.303)**	1.209 (1.123–1.318)**	1.203 (1.184–1.311)**
Emphysema according to CT cut-off RV-950 >5%	41	1.157 (1.075–1.245)**	1.141 (1.054–1.235)*	1.146 (1.055–1.245)*
Airflow obstruction present according to FEV_1_/VC <0.7	38	1.170 (1.088–1.258)**	1.166 (1.083–1.256)**	1.132 (1.044–1.227)*
Airflow obstruction present according to FEV_1_/VC <LLN	36	1.196 (1.109–1.289)**	1.196 (1.107–1.292)**	1.162 (1.069–1.264)**
Emphysema suggested by D_L, CO_ <2SD	28	1.213 (1.117–1.318)**		
Emphysema according to CT cut-off RV-950 >7%	18	1.019 (1.009–1.029)**		
Any respiratory symptom†	219	NS	1.051 (1.005–1.100)*	NS

Model 1, OR per 10 µm crude, unadjusted model. Model 2, with AiDA adjusted for age, sex, height and weight. Model 3, as Model 2 with additional adjustment for pack-years.

Due to small N, models 2 and 3 are not given for emphysema suggested by _DL, CO_ < 2SD and emphysema by CT cutoff RV-950 <7%.

*P<0.05.**p<0.01.

†That is, cough, phlegm, wheezing or dyspnoea.

AiDA, Airspace dimension assessment with nanoparticles; D_L, CO_, diffusing capacity for carbon monoxide; FEV_1_, forced expiratory flow in 1 s; LLN, lower limit of normal; NS, not significant; *r*_AiDA_, distal airspace radius measured with the AiDA method; RV-950, the relative volume of voxels in lung parenchyma with a Hounsfield Unit value less than -950; VC, vital capacity.

## Discussion

This is the first study where distal airspace radii have been determined by nanoparticles in subjects with emphysema. In a previous study, we showed nanoparticle recovery at a single breath-hold time to be different between healthy subjects and patients with moderate to severe COPD. The present study in a population-based sample extends the information to calculation of distal airspace radius, *r*_AiDA_, in subjects with mainly mild emphysema. Our results are in line with comparative methods^7–9^ (online supplemental 5).

10.1136/thoraxjnl-2020-214523.supp5 Supplementary data

The small airways, <2 mm in diameter, have been suggested as the major site of early pathology in COPD. The repetitive toxic deposition stimulates an inflammatory response, repair and remodelling sequence, which later gives rise to a quantifiable airflow obstruction currently used as the diagnostic standard.^10^ There is a long clinically silent period, where the pathophysiological changes do not result in airflow obstruction, and therefore the early stages of COPD often remain undiagnosed.^1^ Also, spirometry alone will not differentiate between obstruction caused by airway narrowing and emphysema.

Due to their small size, nanoparticles traverse the distal airspaces and deposit there by diffusion. The *r*_AiDA_ in healthy volunteers is relatively constant at lung depths between 1000 and 2500 mL.^5^ The *r*_AiDA_ cannot be taken to represent any specific airway generation, but corresponds to a mean of airspaces distal to generation 15–17. This may not apply in diseased airspaces with altered flow; further studies are needed.^5^

AiDA has similarities with *D*_L, CO_, both being dependent on distribution of inhaled gas and diffusion within the airways. In contrast, AiDA is independent of transfer across the air-blood interface, haemoglobin concentration, recent smoking and altitude. The instrument is potentially simpler, as no compressed gases are needed. Compared with CT, the AiDA test is potentially easier and cheaper to administer. AiDA entails neither radiation nor an image that needs interpretation.

The AiDA measurements cause a low exposure to nanoparticles. The subjects were exposed to 0.05% of daily mass and 0.60% of daily particle number exposure in a comparatively clean urban setting.^11^

The study has several limitations. AiDA is a new technology, and we rely on a prototype of the apparatus. The proportion of measurements not fulfilling the technical criteria was high (online supplemental 1). This was mainly caused by the fact that at the beginning of the experiment, the particle concentration in the reservoir, and therefore, the inspired gas, was not uniform, resulting in several insufficient measurements. The subjects without emphysema in this study did not necessarily have normal lungs—a number of subjects had airflow obstruction. Due to the low number of subjects in the population with emphysema, the findings were not further analysed in subgroups according to disease severity, phenotype or presence of bullae. As emphysema and bronchial abnormalities frequently coincide in COPD, it is difficult to examine each phenotype on its own. Further studies on persons with predominantly airway involvement versus parenchymal disease phenotype are warranted, as well as studies to visualise where exactly the particles deposit.

We suggest AiDA is a potential biomarker for emphysema.^1^ To validate the method, however, a diagnostic accuracy study in target populations should be conducted, and sensitivity and specificity calculated.
