# Supplementary material for: Airspace dimension assessment with nanoparticles as a proposed biomarker for emphysema
Source: Thorax. 2021 Apr 15;76(10):1040–3. doi: 10.1136/thoraxjnl-2020-214523 (PMC8461447; doi:10.1136/thoraxjnl-2020-214523)
Supplement: Supplementary data [file thoraxjnl-2020-214523supp001.pdf]

**Online supplement 1: Method and Results**

Material and Method

*Study population*

The study was conducted according to the Declaration of Helsinki. Local ethical board approval was obtained, and written informed consent was obtained from all participants. The study participants completed a questionnaire including medical history, symptoms, and smoking habits. See the questionnaire in Online Supplement 3.

*Technical quality control*

AiDA is a new technique, and the procedures for quality control are under development. In this study, breathing airflow monitor was set to zero before each measurement. The flow and particle size were calibrated monthly. Measurements performed on the same biocontrol individuals before, during, and after the study showed the $r_{\mathrm{AiDA}}$ measurements had a standard deviation below 7 µm. (5) Data were considered acceptable if 1) the subjects performed at least four valid breathing manoeuvres, 2) no instrumental errors were detected, and 3) the correlation coefficient, r, between breath-hold time and log recovery was higher than 0.9, showing sufficient fit to model.

*Computed tomography (CT)*

All chest CTs in this study were performed using the same multidetector-row scanner (Siemens Somatom Definition Flash; Siemens Healthineers, Forchheim, Germany) with a detector configuration of 128 × 0.6, tube voltage 120 kV (Care kV off), tube current modulation (CareDose 4D, ref mAs 30), pitch 0.9, rotation time 0.5 seconds. Images were acquired in full inspiration, and then reconstructed according to following kernels B31f, B20f, I30f (SAFIRE level 3), 0.6 mm slice thickness / 0.6 mm increment, as well as B35f 0.6 mm slice thickness / 0.4 mm increment. This protocol applied low radiation dose (Wang et al AJR 2014).

The images were assessed using syngo.via pulmo 3D software version VA. CT-derived total lung capacity TLC (CTV) was calculated as the volumetric sum of the CT voxels containing lung tissue at full inspiration. A density mask of -950 Hounsfield Units (HU) was applied. The percentage of voxels with a HU value below -950, termed relative volume, or RV -950, was applied. Emphysema was then defined using two different RV -950 percentage cutoff-values; > 7% or > 5%. Visually assessed emphysema was

defined by the Fleischner society's guidelines and recorded as present or absent. If present, a semiquantitative score was obtained as described by Goddard et al *Computed tomography in pulmonary emphysema. Clinical radiology 1982*; each lung was divided into an upper, middle and lower section, and for each section an emphysema score between 0 and 3 was assigned, where 0 represented no emphysema, 1 represented 0-25% emphysema, 2 denoted a 25-50% emphysema and 3 represented above 50% emphysema. The scores from each section were added together, yielding a maximum score of 18.

*Statistics*

Data were examined for skewness and kurtosis to test normality. Student's t-test was used to determine differences in in study variables between emphysematous and non-emphysematous subjects.

Three logistic regression models were used to investigate differences in $r_{AiDA}$ odds ratios between emphysematous and non-emphysematous subjects, as well as between persons with and without airflow obstruction. Three different definitions of emphysema were used in the logistic regression: visually detected emphysema, RV -950 above 5% and RV-950 above 7%. Airflow obstruction was defined both according to GOLD criteria, as well as using a cut-off of $FEV_1/VC < 1.65$ SD, i.e. lower limit of normal. Lastly, a cutoff in $D_{L,CO}$ of -2SD was used. In model 1, each variable was considered alone without adjusting for other factors. In model 2, age, BMI and sex were accounted for. In model 3, age, BMI, sex as well as pack years were adjusted for.

Additionally, to avoid overfitting of the logistic regression models, we also used the residual method to adjust the AiDA values for confounding factors (Online supplement 1, Table 3). A multiple linear regression model was used. AiDA (dependent variable) was regressed on age, sex, height and weight (independent) and the residual values were saved. The residuals were normally distributed. In the next step, three logistic regression models were used to examine the relationships between $r_{AiDA}$ and various definitions of emphysema, including CT, airflow obstruction and reduced $D_{L,CO}$. Model 1 was unadjusted. In model 2, residuals of AiDA (adjusted for age, height, weight and sex) were entered. were accounted for and in model 3, pack years was added to the model as a separate variable.

In each analysis, missing data were reported. Student's t-test was used to compare emphysema/airflow obstruction indices between included subjects and subjects excluded due to insufficient AiDA measurements. Student's t-test was also used to determine differences in $r_{AiDA}$ with respect to the comorbidities listed under Online Supplement 3.

A p-value of less than 0.05 was considered statistically significant. The analyses were conducted using IBM SPSS (v. 24, 2016, Armonk, NY).

*Study size*

Past literature on AiDA is scarce. A power calculation was made based on data from a proof-of-concept study. Assuming a COPD prevalence of 10%, a sample size of 104 individuals is required (two-tailed $\alpha$ = 0.05; $\beta$ = 0.2) to establish sufficient power to prove a difference between emphysematous and non-emphysematous subjects. As the proof-of-concept study included subjects with advanced disease, and only mild emphysema was expected in a population-based sample, a sample size 5-6 times the above calculation was obtained.

## Results

**Supplement Table: –Logistic regression models of $r_{AiDA}$ and measures of emphysema, with adjustments for covariates using the residual method. Odds ratios (95% confidence intervals) N=618**

| | N | Model 1 OR | Model 2 OR | Model 3 OR |
|---|---|---|---|---|
| Emphysema present in CT, visual evaluation | 47 | 1.216 (1.134-1.303)** | 1.209 (1.127-1.298)** | 1.163 (1.082-1.249)** |
| Emphysema according to CT cutoff RV-950 > 5% | 41 | 1.157 (1.075-1.245)** | 1.135 (1.054-1.223) * | 1.144 (1.057-1.237)* |
| Airflow obstruction present according to $FEV_1/VC$ < 0.7 | 38 | 1.170 (1.088-1.258)** | 1.163 (1.081-1.252)** | 1.130 (1.047-1.219)** |
| Airflow obstruction present according to $FEV_1/VC$ < LLN | 36 | 1.196 (1.109-1.289)** | 1.192 (1.105-1.286)** | 1.161 (1.073-1.255)** |
| Emphysema suggested by $D_{L,CO}$ < 2SD | 28 | 1.213 (1.117-1.318)** | | |
| Emphysema according to CT cutoff RV-950 > 7% | 18 | 1.019 (1.009-1.029)** | | |
| Any respiratory symptom‡ | 219 | NS | 1.055(1.008-1.104) * | NS |

Model 1, odds ratio per 10 µm crude, unadjusted model. Model 2, with AiDA adjusted for age, sex, height and weight (residual method). Model 3, as Model 2 with additional adjustment for pack years.

Abbreviations: CT, computed tomography RV-950; the relative volume of voxels in lung parenchyma with a Hounsfield Unit value less than -950; $FEV_1$, forced expiratory flow in one second; VC, vital capacity; LLN, lower limit of normal; $D_{L,CO}$, diffusing capacity for carbon monoxide; $r_{AiDA}$, distal airspace radius measured with the AiDA method; NS, not significant, OR odds ratio.
*P<0.05
**P<0.0001

‡ I.e. cough, phlegm, wheezing or dyspnea

Due to small N, models 2 and 3 are not given for emphysema suggested by $D_{L,CO}$ < 2SD and emphysema according to CT cutoff RV-950
> 7%.

**Supplement figure – Residual distribution**

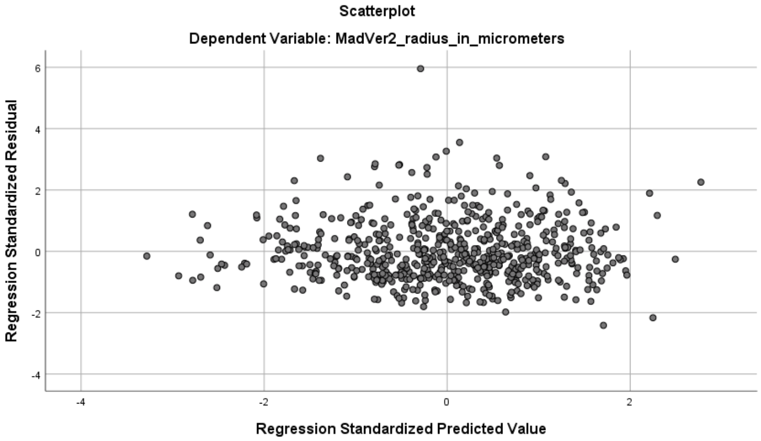

The persons excluded due to insufficient quality of AiDA measurements did not differ from the included with respect to age, $FEV_1$, $D_{L,CO}$ and RV-950.
