# Supplementary material for: Airspace dimension assessment with nanoparticles as a proposed biomarker for emphysema
Source: Thorax. 2021 Apr 15;76(10):1040–3. doi: 10.1136/thoraxjnl-2020-214523 (PMC8461447; doi:10.1136/thoraxjnl-2020-214523)
Supplement: Supplementary data [file thoraxjnl-2020-214523supp002.pdf]

# Online Supplement 2: Questionnaire

## Respiratory symptoms

In this paper, self-reported respiratory symptoms were defined as an affirmative answer to any of the following questionnaire items: “Do you cough even when you do not have a common cold?”, “Do you cough up phlegm, or experience mucus in your chest that you have trouble clearing, even when you do not suffer from a common cold?” “Do you experience wheezing or whistling in your lungs?”, “Do you experience shortness of breath when hurrying on level ground or walking up a slight incline?”, or “Have you ever suffered from shortness of breath severe enough to disturb your daily activities, or to force you to stay home from work?”

## Comorbidities

The subjects were given the following questions regarding their comorbidities.

Which of the following illnesses has a medical doctor diagnosed on you, or have been surgically treated for? (several alternatives can be chosen)

- Myocardiac infarct/a thrombus in the heart*
- Angina pectoris*
- Atrial fibrillation*
- Heart failure*
- Valvular heart disease*
- A bypass-operation or balloon dilatation of the coronary arteries*
- A procedure for treating stenosis caused by peripheral artery disease*
- A procedure for aortic aneurysm*
- Stroke/brain infarct/bleeding in the brain*
- High blood pressure
- High blood lipids / high cholesterol
- Diabetes
- Chronic obstructive pulmonary disease
- Asthma

- Tuberculosis
- Other lung disease
- Obstructive sleep apnea
- Gluten intolerance/coeliac disease
- Crohn's disease or ulcerative colitis
- Rheumatic disease
- Cancer
- None of the above

Cardiovascular disease other than hypertension is denoted by *
