# Supplementary material for: Airspace dimension assessment with nanoparticles as a proposed biomarker for emphysema
Source: Thorax. 2021 Apr 15;76(10):1040–3. doi: 10.1136/thoraxjnl-2020-214523 (PMC8461447; doi:10.1136/thoraxjnl-2020-214523)
Supplement: Supplementary data [file thoraxjnl-2020-214523supp004.pdf]

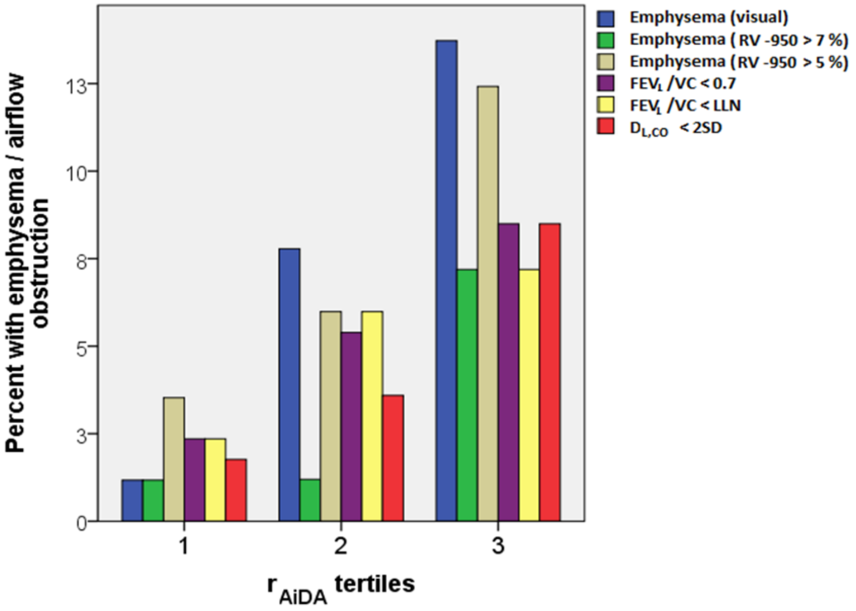

Online Supplement 4: The percentage of subjects with different emphysema and airflow obstruction indices and $D_{L,CO}$ < 2 SD.
The subjects are divided into tertiles according to increasing r_{AiDA} values. For each variable, emphysema or outflow obstruction is considered either present or absent. The percentage of present findings per r_{AiDA} tertile are given.
$D_{L,CO}$ = diffusing capacity for carbon monoxide, SD = standard deviation, RV -950 = percent of voxels with an attenuation value below -950 Hounsfield Units, FEV_1 = forced expiratory flow in one second, VC = vital capacity, LLN = lower limit of normal, r_{AiDA} = distal airspace radius measured using Airspace Dimension Assessment with nanoparticles
