# Supplementary material for: Airspace dimension assessment with nanoparticles as a proposed biomarker for emphysema
Source: Thorax. 2021 Apr 15;76(10):1040–3. doi: 10.1136/thoraxjnl-2020-214523 (PMC8461447; doi:10.1136/thoraxjnl-2020-214523)
Supplement: Supplementary data [file thoraxjnl-2020-214523supp003.pdf]

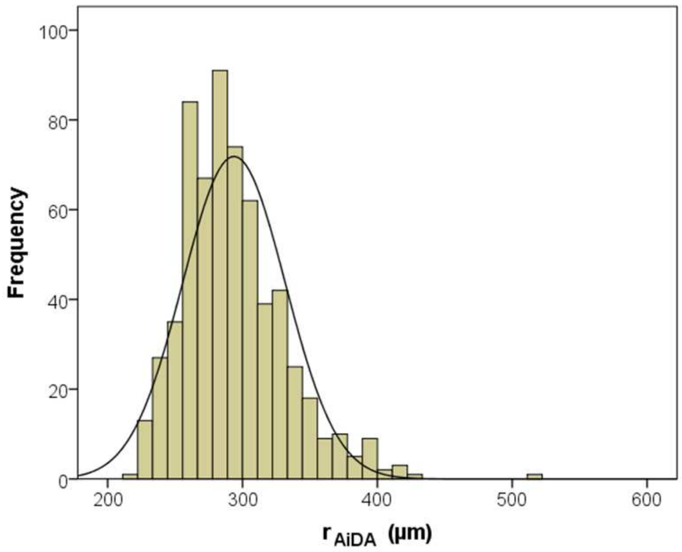

Online Supplement 3 - Frequency table of rAiDA with a normal distribution curve
The AiDA values were approximately normally distributed. There was one female outlier value with r_{AiDA} of 516 mm. This person had moderate emphysema on CT with a visual emphysema score of 8 (of 18). She had COPD stage 2 according to spirometric GOLD-criteria (FEV_1/VC < 0.7 and FEV_1 of 55% of predicted). She also had a $D_{L,CO}$ of 49% of predicted. This person was an active smoker with a >52 pack year smoking history. She also reported experiencing dyspnoea, cough, wheezing and increased phlegm.
