# Supplementary material for: Airspace dimension assessment with nanoparticles as a proposed biomarker for emphysema
Source: Thorax. 2021 Apr 15;76(10):1040–3. doi: 10.1136/thoraxjnl-2020-214523 (PMC8461447; doi:10.1136/thoraxjnl-2020-214523)
Supplement: Supplementary data [file thoraxjnl-2020-214523supp005.pdf]

Online Supplement 5: Distal airspace diameter measured by different methods

STUDY	Method	Site of measurement	Number of subjects	Average diameter, non-emphysematous lung	Average diameter, emphysematous lung	Comment
Tanabe 2017	Micro-CT	Lm	Controls N = 7<br>CLE N = 6<br>PLE N = 7	336 ± 37 µm	CLE: 766 ± 259 µm<br>PLE: 698 ± 240 µm	
Woods 2006	^{3}He MRI	Lm	Controls N = 6<br>COPD N = 6	200 µm	410 µm	No SD given.
Kohlhaufl 1999	ADAM	Distal airspaces	Non-emphysematous N = 30<br>Emphysematous N = 20	330 ± 100 µm	840 ± 530 µm	
The present study	AiDA	Distal airspaces	Non-emphysematous N = 563<br>Emphysematous N = 47	582 ± 72 µm	652 ± 96 µm	Diameter = 2 x radius.

CT = computed tomography, CLE = centrilobular emphysema, PLE = panlobular emphysema, ^{3}He MRI = hyperpolarised helium magnetic resonance imaging, Lm = mean linear intercept, a measure of alveolar/acinar structures, ADAM = aerosol derived airway morphometry, AiDA = airspace dimension assessment with nanoparticles
